# Protective effects of long-term administration of *Ziziphus jujuba* fruit extract on cardiovascular responses in L-NAME hypertensive rats 

**Published:** 2018

**Authors:** Reza Mohebbati, Kosar Bavarsad, Maryam Rahimi, Hasan Rakhshandeh, Abolfazl Khajavi Rad, Mohammad Naser Shafei

**Affiliations:** 1 *Department of Physiology, Faculty of Medicine, Mashhad University of Medical Sciences, Mashhad, Iran*; 2 *Pharmacological Research Center of Medicinal Plants, Mashhad University of Medical Sciences, Mashhad, Iran *; 3 *Neurogenic Inflammation Research Centre, Mashhad University of Medical Sciences, Mashhad, Iran*

**Keywords:** Ziziphus jujube, Nitro-L-arginine methyl ester, Blood pressure, Hypertension, Nitric oxide

## Abstract

**Objective:**

*Ziziphus jujuba *stimulates the release of nitric oxide (NO). Because NO is involved in cardiovascular regulations, in this study the effects of hydroalcoholic extract of *Z. jujuba* on cardiovascular responses in acute NG-nitro-L-arginine methyl ester (L-NAME) hypertensive rats were evaluated.

**Materials and Methods:**

Rats were divided into 6 group (n=6): 1) saline, 2) L-NAME received (10mg/kg) intravenously, 3) sodium nitroprusside (SNP) (50µg/kg)+L-NAME group received SNP before L-NAME and 4-6) three groups of *Z. jujuba* (100, 200 and 400mg/kg) that treated for four weeks and on the 28^th^ day, L-NAME was injected. Femoral artery and vein were cannulated for recording cardiovascular responses and drug injection, respectively. Systolic blood pressure (SBP), Mean arterial pressure (MAP) and heart rate (HR) were recorded continuously. Maximal changes (∆) of SBP, MAP and HR were calculated and compared to control and L-NAME groups.

**Results:**

In L-NAME group, maximal ΔSBP (L-NAME: 44.15±4.0 mmHg vs control: 0.71±2.1 mmHg) and ΔMAP (L-NAME: 40.8±4.0 mmHg vs control: 0.57±1.6 mmHg) significantly increased (p<0.001 in both) but ∆HR was not significant as compared to control (p>0.05). All doses of *Z. jujuba* attenuated maximal ∆SBP and ∆MAP induced by L-NAME but only the lowest dose (100 mg/kg) had significant effects (ΔSBP: 20.36±5.6 mmHg vs L-NAME: 44.1±4.0 mmHg and ΔMAP: 20.8±4.5 mmHg vs L-NAME: 40.8±3.8 mmHg (p<0.05 to p<0.01)). The ∆HR at three doses was not significantly different from that of L-NAME group (p>0.05).

**Conclusion:**

Because long-term consumption of *Z. jujuba* extract, especially its lowest dose, attenuated cardiovascular responses induced by L-NAME, we suggest that *Z. jujuba* has potential beneficial effects in prevention of hypertension induced by NO deficiency.

## Introduction

Nitric oxide (NO) is an active gaseous molecule that plays an important role in regulation of regional blood flow, blood pressure, platelet aggregation, vascular smooth muscle proliferation and mediator of nociception in acute and chronic pain conditions (Naseem, 2005 ▶; Sasser et al., 2011 ▶; Abbasnezhad et al., 2016 ▶). NO is synthetized from L-arginine by three isoforms (neuronal, inducible and endothelial) of nitric oxide synthases (NOS). From these isoforms, endothelial NOS (eNOS) is mostly involved in synthesis of NO in endothelium and has protective effect on cardiovascular system. Endothelium dysfunction results in decreased NO bioavailability and leads to several cardiovascular problem including development of hypertension (Naseem, 2005 ▶). In addition, agents that inhibit NOS activity increase cardiovascular responses. For example NG-nitro-L-arginine methyl ester (L-NAME), a NOS inhibitor induces hypertension by inhibition of NO synthesis, in animals (Khayyal et al., 2002 ▶). In addition, agents that increase NO bioavailability may potentially have therapeutic uses in hypertension treatment (Sasser et al., 2011 ▶). 

*Ziziphus jujuba (Z. jujuba) is* a plant belonging to the Rhamnaceae family. *Z. jujuba* has a great history of usage both as a remedy and a fruit (Mahajan and Chopda, 2009 ▶). The main biologically active components of plant are vitamins C and E, flavonoids, phenols, triterpene acids, polysaccharides and saponins (Cheng et al., 2000 ▶; Gao et al., 2013 ▶). Recent pharmacological studies showed that *Z. jujuba* has many pharmacological effects such as anticancer (Huang et al., 2007 ▶), anti-inflammatory (Al-Reza et al., 2010 ▶), antioxidant (Zhang et al., 2010 ▶), hepatoprotective (Wang et al., 2012 ▶) and many other protective activities in organs and tissues. Hypotensive effect of *Z. jujuba* has been reported to be mediated via stimulation of the release of NO (Kim and Han, 1996 ▶). The hypotensive effect of *Z. jujuba* also has been reported previously (Mahajan and Chopda, 2009 ▶). Involvement of several compound of *Z. jujuba* such as jujuboside, saponins in cardiovascular regulation has also been shown (Steinkamp-Fenske et al., 2007 ▶; Zhao et al., 2016 ▶). However, the exact mechanism underlying the effect of *Z. jujuba* on cardiovascular system is yet to be determined. Because *Z. jujuba* stimulates the release of NO *in vitro* (in cultured endothelial cells) and *in vivo* (Kim and Han, 1996 ▶), it is possible that cardiovascular effect of *Z. jujuba* is mediated by NO. To determine if cardiovascular effect of *Z. jujuba* is mediated via NO system, the cardiovascular effects of *Z. jujuba* were assessed in acute L-NAME-treated hypertensive rats (Khayyal et al., 2002 ▶). 

## Materials and Methods

**Extract preparation**

 Fruits of *Z. jujuba* were provided from herbs store, Birjand, Iran, and identified by botanists in Herbarium of Ferdowsi University of Mashhad. Then, 100 g of dried fruit without seed was powdered then macerated in 1000 ml ethanol 70% and shaked for 72 hr. After that, the mixture was filtered through different filter sizes. The solvent was evaporated by an oven at 40^°^ C (Mohebbati et al., 2016 ▶). The yield percentage of Z. jujuba was 60%. Different concentrations of the *Z. jujuba* fruit extract were prepared by adding saline.

**Animals and surgery**

 Forty-two male Wistar rats were used in this study. The animals were anesthetized using urethane (1.5 g/kg, i.p). Animal temperature was kept at 37 °C with a heating lamp. The left femoral artery was cannulated with a polyethylene catheter (PE-50) filled with heparinized saline then catheter was connected to a blood pressure transducer and blood pressure (BP) and heart rate (HR) were continuously recorded by a Power Lab system (ID instrument, Australia) (Shafei and Nasimi, 2011 ▶). The right femoral vein was also cannulated for drug injection. 

**Experimental protocol**

The L-NAME group received L-NAME (10mg/kg) intravenously (i.v) (Hu et al. 1997 ▶), in sodium nitroprusside (SNP) group, SNP (50mg/kg, i.v) (Hirschl et al., 1997 ▶) was injected 5 min before L-NAME. In the *Z. jujuba* groups, rats were treated with three doses of hydroalcoholic extract of *Z. jujuba* (100, 200 and 400 mg/kg) (Goyal et al., 2011 ▶) by gavage for four week. On day 28, the L-NAME (10 mg/kg, i.v) was injected. In all groups, systolic blood pressure (SBP), mean atrial pressure (MAP) and heart rate (HR) were recorded throughout the trial period.

**Drug and animal groups**

The drugs including urethane, L-NAME and SNP were purchased from Sigma, USA. All drugs were dissolved in saline.

Rats were randomly divided into 6 groups as follow (n=7 in each group)

1. Control group received saline (i.v).

2. L-NAME group received L-NAME (10mg/kg, i.v).

3. SNP group received SNP (50µg/kg, i.v) before injection of L-NAME 10mg/kg (i.v).

4. *Z. jujuba* 100 group orally received 100 mg/kg of extract for four weeks and on day 28, they received L-NAME 10mg/kg (i.v). 

5. *Z. jujuba* 200 group orally received 200 mg/kg of extract for four weeks and on day 28 received L-NAME 10mg/kg (i.v).

6. *Z. jujuba* 400 group orally received 400 mg/kg of extract for four weeks and on day 28 received L-NAME 10mg/kg (i.v).

**Statistical analysis**

Changes (∆) in MAP, SBP and HR values were calculated and expressed as mean±SEM. Statistical comparisons were done by one-way ANOVA followed by the Tukey’s *post hoc* test. A p<0.05 was considered statistically significant.

## Results

**Effects of saline on cardiovascular responses**

After a stabilizing time of 10 min, saline was injected intravenously and cardiovascular responses were recorded. Injection of saline had no significant effects on SBP (before: 122±10 mmHg and after: 123±10 mmHg; p>0.05), MAP (before: 114±10 mmHg and after: 115±10 mmHg; p>0.05) or HR (before: 334±16 beats/min and after: 336±22 beats/min; p>0.05).

**Effect of intravenous injection of L-NAME alone and after pre-treatment with SNP on cardiovascular responses**

**Figure 1:**
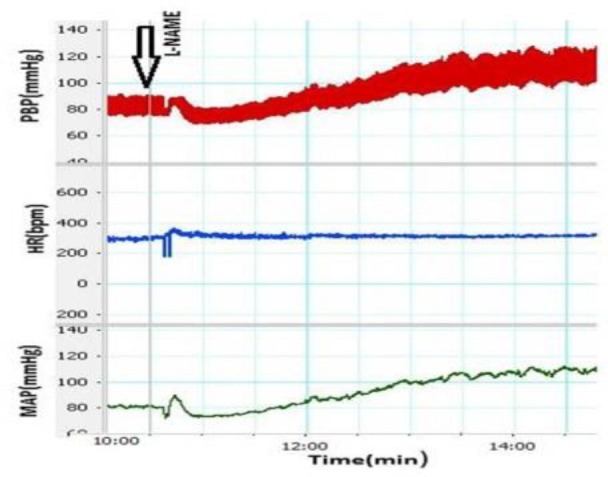
A sample of recording of cardiovascular parameter after i.v injection of L-NAME

**Figure 2:**
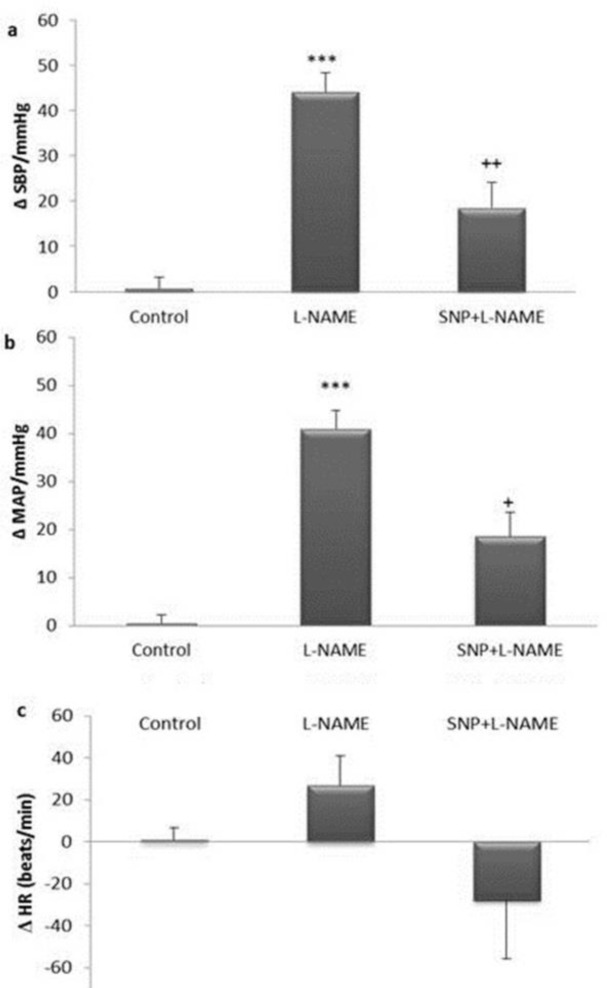
Effects of L-NAME (10mg/kg; i.v) and L-NAME+SNP (50 µg/kg) on ΔSBP (a), ΔMAP (b) and ΔHR (c) in anesthetized rats (n=6).

To evaluate the effects of L-NAME, L-NAME alone (10 mg/kg; i.v) was slowly injected and cardiovascular responses (SBP, MAP and HR) were recorded (Figure 1). Mean ∆SBP and ∆MAP after injections of L-NAME are shown in Figure 2a and b. It was observed that ∆SBP and ∆MAP significantly increased compared to control group (ΔSBP: L-NAME group 44.15±4.0 mmHg vs control group: 0.71±2.1 mmHg (p<0.001) and ΔMAP:L-NAME group (40.8±4.0 mmHg vs control group: 0.57±1.6 mmHg (p<0.001)). The HR also increased compared to control group but it was not significant (ΔHR: L-NAME group 26.6±14 vs control group 1±4.9 beats/min (p>0.05)) (Figure. 2 c).

The data were compared with control group and expressed as mean ± SEM. One-way ANOVA was used for statistical analysis. *** p<0.001 compared to control.+ p<0.05 and ++ p<0.01 compared to L-NAME.

ΔSBP: Changes of systolic blood pressure, ΔMAP: Changes of mean arterial pressure and ΔHR: Changes of heart rate

In SNP group, SNP was injected (50 µg/kg, i.v) before L-NAME. SNP ameliorated increased cardiovascular responses induced by L-NAME. Figure 2 a and b show the effect of SNP on SBP and MAP. Based on our results, pre-treatment with SNP could significantly attenuate the effect of L-NAME on cardiovascular responses (ΔSBP in SNP+L-NAME group: 18.6±5.5 mmHg vs ΔSBP in L-NAME group: 44.1±4.0 mmHg (p<0.01) and ΔMAP in SNP+L-NAME group: 18.5±5.1 mmHg vs ΔMAP in L-NAME group: 40.8±3.8 mmHg (p<0.05). The changes in HR in SNP+L-NAME group were also lower compared to L-NAME group but the difference was not significant (ΔHR in SNP+L-NAME group: -27.9±27.7 vs ΔHR in L-NAME group: 26.6±14 beats/min (p>0.05) (Figure 1c). The HR changes in SNP+L-NAME group was also not significantly different from those of control group (p>0.05). 

**Effect of hydroalcoholic extract of** ***Ziziphus jujuba*** **fruits on cardiovascular responses in L-NAME hypertensive rats**

**Figure 3:**
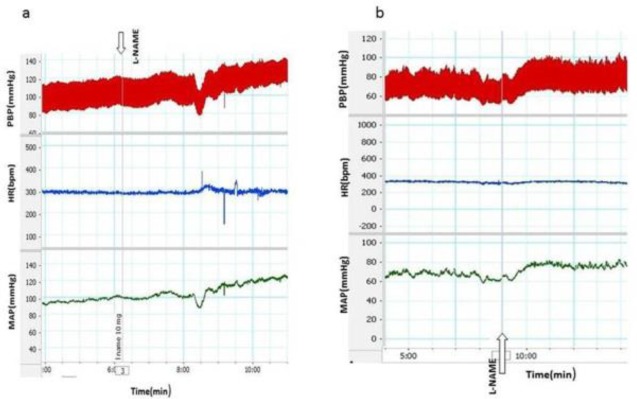
Samples of recording of cardiovascular parameter induced by injection of L-NAME after pretreatment with two doses100 mg/kg (a) and 400 mg/kg(b) of Z. jujube

**Figure 4:**
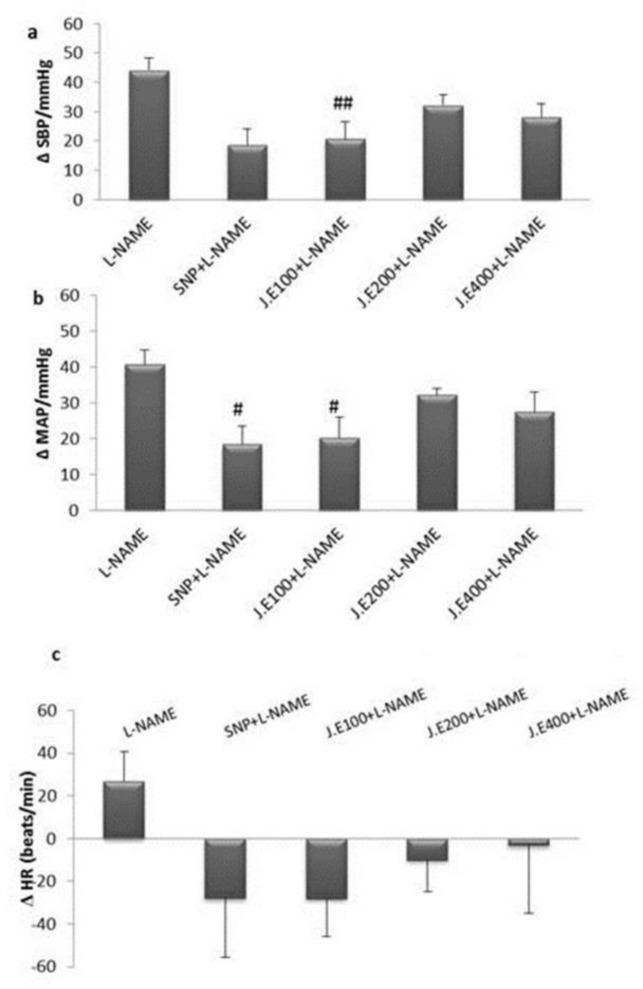
Effects of hydroalcoholic extract of *Z. jujuba* on cardiovascular responses in L-NAME hypertensive rats (n= 6).

Rats treated with three doses of *Z. jujuba* (100, 200, and 400 mg/kg, orally), for 4 weeks, then received L-NAME (10 mg; i.v) slowly on day 28 and cardiovascular responses were recorded( Figures 3). In *Z. jujuba* 100mg/kg +L-NAME group, ΔSBP and ΔMAP significantly decreased compared to L-NAME group (ΔSBP for *Z. jujuba* 100mg/kg +L-NAME: 20.36±5.6 mmHg vs ΔSBP for L-NAME: 44.1±4.0 mmHg and ΔMAP for *Z. jujuba* 100mg/kg +L-NAME: 20.8±4.5 mmHg *vs* ΔMAP for L-NAME: 40.8±3.8 mmHg (p<0.05 to p<0.01) (Figure. 4 a and b). Changes in HR at this dose (100mg/kg) was not significantly different from those of L-NAME alone (ΔHR for *Z. jujuba* 100mg/kg +L-NAME: 28.1±17.8 vs ΔHR for L-NAME: 26.6±14.0, beats/min (p>0.05)) (Figure 4 c). Changes in all responses at this dose were not significantly different from those of SNP+ L-NAME group.

Rats were treated with three doses of *Z. jujuba* (100, 200 and 400mg/kg) for four weeks then, L-NAME was injected and cardiovascular responses were determined. The data from *Z. jujuba-*treated rats were compared with those of L-NAME group and expressed as mean±SEM. One-way ANOVA was used for statistical analysis.

# p<0.05 and ## p<0.01 compared to L-NAME

SBP: Systolic blood pressure, MAP: mean arterial pressure, and HR: heart rate

In rats treated with *Z. jujuba* 200 mg/kg+L-NAME, ΔSBP and ΔMAP did not significantly reduce compared to L-NAME group (ΔSBP in *Z. jujuba* 200 mg/kg+L-NAME group: 32.2±3.5 vs ΔSBP in L-NAME group: 44.1±4.0 mmHg (p>0.05) and ΔMAP in *Z. jujuba* 200 mg/kg+L-NAME group: 32.2±1.6 mmHg vs ΔMAP in L-NAME group: 40.8±3.8 mmHg (p>0.05)) (Figure 4a and b). Changes in HR at this dose (200 mg/kg) were not significantly different from those of L-NAME group (ΔHR in *Z. jujuba* 200 mg/kg+L-NAME group: -10±15.0 vs ΔHR in L-NAME group: 26.6±14.0, beats/min (p>0.05) (Figure 4c). Changes in all responsess at this dose were not significantly different from those of SNP + L-NAME group.

In rats treated with *Z. jujuba* 400 mg/kg+L-NAME, ΔSBP and ΔMAP were lower than L-NAME group but were not significantly different (ΔSBP in* Z. jujuba* 400 mg/kg+L-NAME 28.1±4.5 vs ΔSBP in L-NAME group: 44.1±4.0 mmHg (p>0.05) and ΔMAP in* Z. jujuba* 400 mg/kg+L-NAME: 27.5±5.4 mmHg vs ΔMAP in L-NAME group: 40.8±3.8 mmHg (p>0.05)) (Figure 4 a and b). Changes in HR at this dose (400 mg/kg) were also not significantly different from those of L-NAME group. (ΔHR in* Z. jujuba* 400 mg/kg+L-NAME: -3.1±31.7 vs ΔHR in L-NAME group: 26.6±14.0 beats/min (p>0.05)) (Figure 4c ). Changes in all responses at this dose (400 mg/kg) were not significantly different from those of SNP+L-NAME group.

**Effect of hydroalcoholic extract of** ***Ziziphus jujuba*** **on body weight**

**Figure 5:**
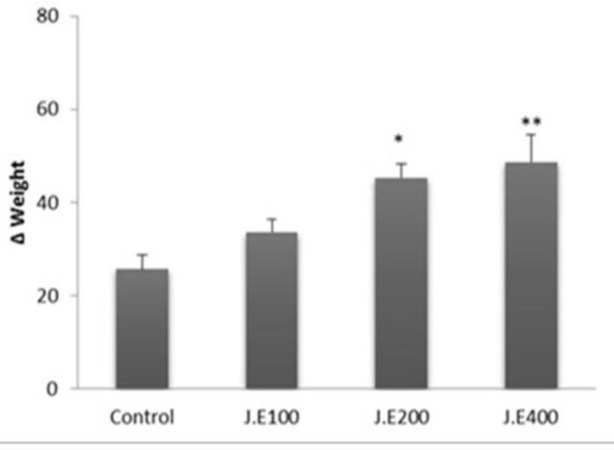
Effects of hydroalcoholic extract of *Z. jujuba* on body weight of rats after four week treatment (n=6). One-way ANOVA was used for statistical analysis.

The body weight in all animals treated with the extract increased. However, *Z. jujuba* extract at 200 and 400 mg/kg significantly increased rats body weight compared to control group (p<0.05 to p<0.01; Figure 5).

* p<0. 05 and ** p<0.01 compared to control .

## Discussion

In the present study, the effects of sub-chronic administration of *Z. jujuba* fruits hydroalcoholic extract to L-NAME hypertensive rats investigated.

Our results showed that four-week administration of three doses of *Z. jujuba* (100, 200 and 400 mg/kg) ameliorates L-NAME-induced hypertension.

NO, is a potent vasodilator that has an important role in cardiovascular regulation (Fadel, 2017 ▶). The vasodilatory effect of NO is mediated by increased production of guanosine 3′, 5′-cyclic monophosphate in vascular smooth muscle (Fadel, 2017 ▶). It is well known that inhibition of NO production is associated with increased blood pressure and induction of hypertension (Zicha et al., 2006 ▶). In the present study, we used L-NAME, a well-known NOS inhibitor for inhibition of NO production (Zicha et al., 2006 ▶). Our result indicated that L-NAME could increase MAP, SBP with a mild effect on HR that is consistent with previous studies (Khayyal et al., 2002 ▶). We observed that L-NAME at the dose of 10 mg/kg did not have a significant effect on HR. The pharmacological vasodilators, nitroglycerin and SNP both cause vasodilation by donation of exogenous NO or NO-like compounds (Mohebbati et al., 2016a). In this study, injection of SNP before L-NAME ameliorated L-NAME-induced hypertension that confirms involvement of NO in cardiovascular regulation (Jerkic et al., 2004 ▶). HR in SNP+L-NAME group, was also lower than baseline value which might be due to direct effect of NO released from SNP on nodes of heart or promotion of parasympathetic activity (Klimaschewski et al., 1992 ▶). The ameliorative effects of different doses of Z. jujuba on cardiovascular responsess in hypertension induced by L-NAME suggest NO involvement in cardiovascular effects of *Z. jujuba* fruits extract. Consistent with our results, a previous study reported that *Z. jujuba* stimulates the release of NO in cultured endothelial cells (Kim and Han, 1996 ▶). Therefore, it is conceivable that this effect of extract might be mediated via affecting endothelium of vessels and increasing NO production. In addition, there is evidence that sympathetic activity is increased in L-NAME hypertension (Biancardi et al., 2007 ▶) and it is conceivable that the effect of extract is mediated via inhibition of sympathetic nervous system. It has also been reported that NO under normal condition, inhibits the release of endothelin-1 from endothelium. Therefore, after blockade of NO in acute L-NAME hypertension, release of endothelin increased and caused vasoconstriction (Banting et al., 1992 ▶). It is possible that *Z. jujuba* antagonizes endothelin-1 receptors and decreases blood pressure. However, future studies are needed to clarify this hypothesis.

 Our results also showed that the best effect of the extract was achieved at the lowest dose (100 mg/kg). The mechanism of this effect is unknown; but, it is possible that doses that we used in this study are high. Therefore, the lowest dose could induce the maximum vasorelaxant effect. In addition, endothelium beside production of vasorelaxant agents produces vasoconstrictor agents such as thromboxane A2 and prostaglandins (Kato et al., 1990 ▶). It is possible that at higher dose (400mg/kg), the extract activates vasoconstrictor agents and by amelioration of vasorelaxant effects of NO, it could increase cardiovascular responses. 

The existence of active biological compounds such as flavonoids, phenols, alkaloids, terpenoids and vitamins has been shown in *Z. jujuba* (Cheng et al., 2000 ▶; Fisher et al., 2003 ▶). Many of these compounds affect NO production or show protective effects on cardiovascular system. For example in our previous study, *Trigonella foenum *plant could increase NO production in an endothelial cell line. This effect of *T. foenum* was mostly induced by diosgenin, which also named sapogenin (Mohebbati et al., 2016a ▶). As sapogenin has also been isolated from *Z. jujuba*, it is conceivable that this compound and its derivative are involved in cardiovascular effect of *Z. jujuba* by increasing NO production*. *Jujuboside is another active compound of *Z. jujuba* that can reduce the vascular tone by activation of NOS (Zhang et al., 2002 ▶; Zhao et al. 2016 ▶). Several studies showed that flavonoid (Freedman et al., 2001 ▶) compounds found in many herbs including *Z. jujuba* cause vasodilation via increment of endothelial NO production. Therefore, effect of *Z. jujuba* on blood pressure may be mediated by flavonoids (Achike and Kwan, 2003 ▶; Han et al., 2001 ▶; Mohebbati et al., 2016 ▶). Betulinic acid is a phenolic compound that has been isolated from *Z. jujuba* (Mahajan and Chopda, 2009 ▶). Betulinic acid also affects eNOS activity (Steinkamp-Fenske et al., 2007), increases bioavailability of NO and has protective effects on cardiovascular system.

There are several evidence showing that increased reactive oxygen species (ROS) production could alter several physiological functions of endothelium including NO production and are involved in pathogenesis of hypertension (Schulz et al., 2011 ▶). Antioxidant agents by improvement of endothelial function and increasing NO levels, have beneficial effect on cardiovascular system. Previous studies have shown antioxidant effect of *Z. jujuba* (Taati et al., 2011 ▶; Zhang et al., 2010 ▶). Therefore, cardiovascular effect of this plant maybe mediated by its antioxidant properties. Inflammation is another important factor involved in cardiovascular diseases and hypertension (Savoia and Schiffrin, 2006 ▶). Anti-inflammatory effect of *Z. jujuba* reported in previous studies (Al-Reza et al., 2010 ▶; Goyal et al., 2011 ▶). The jujubosides, flavonoids and terpenes are important compounds of *Z. jujuba* that have anti-inflammatory activities which may produce beneficial cardiovascular effect of *Z. jujuba.*

Also in rats treated with *Z. jujuba*, body weights increased dose dependently. This increase in weight may be due to appetite increment in rats (Stewart, 2004 ▶). It has been shown that NO is important in regulation of appetite (Morley et al., 2011 ▶). As body weight of animals increased dose-dependently, it is possible that *Z. jujuba* increases animal's appetite through a NO-dependent mechanism.

In summary, a few studies evaluated cardiovascular effect of *Z. jujuba*. Our results for the first time showed that hydroalcoholic extract of *Z. jujuba* attenuates the acute L-NAME hypertension. Therefore, it is conceivable that cardiovascular effect of this extract is mediated by NO production. 

Because all doses of hydroalcoholic extract of *Z. jujuba*, especially the lowest does, could suppress cardiovascular responses induced by L-NAME, we suggest that long-term consumption of this plant has beneficial effects for prevention of hypertension induced by NO deficiency.
